# Sociomoral Reasoning in Adults with ADHD: A Pilot Study

**DOI:** 10.3934/publichealth.2014.3.147

**Published:** 2014-08-07

**Authors:** Kate E. Thomason, Gisli Gudjonsson, Elaine German, Robin Morris, Susan Young

**Affiliations:** 1 Cardiff and Vale UHB, Wales, CF14 4XW, UK; 2 King's College London, Institute of Psychiatry, WC2R 2LS, UK; 3 King's College Hospital, London, SE1 9RT, UK; 4 Imperial College London, SW7 2AZ, UK

**Keywords:** Attention Deficit Hyperactivity Disorder, sociomoral reasoning, antisocial personality, intelligence

## Abstract

Attention Deficit Hyperactivity Disorder (ADHD) is frequently linked with antisocial behaviour, yet less is known about its relationship with sociomoral reasoning, and the possible mediating effect of intelligence. A pilot study was designed to investigate the relationship between antisocial personality traits, intelligence and sociomoral reasoning in adults with ADHD. Twenty two adults with ADHD and 21 healthy controls, matched for age, gender and IQ completed a battery of measures including the National Adult Reading Test, Gough Socialisation Scale and Sociomoral Reflection Measure-Short Form. There was no difference between the groups and levels of sociomoral reasoning, despite the ADHD group reporting greater antisocial personality traits. Sociomoral reasoning was positively correlated with intelligence. Results from a hierarchical multiple regressions indicated that both antisocial traits and IQ were significant predictors of sociomoral reasoning, with IQ proving the most powerful predictor. Whilst antisocial personality traits may explain some of the variance in levels of sociomoral reasoning, a diagnosis of ADHD does not appear to hinder the development of mature moral reasoning. Intellectual functioning appears to facilitate the development of sociomoral reasoning. A further analysis showed that both ADHD and low sociomoral reasoning were significant predictors of antisocial traits. The current findings have important treatment implications.

## 1. Introduction

### 1.1. Sociomoral reasoning

Arguably, the most influential approach to moral development is the cognitive-developmental perspective of Kohlberg [1], who built on Piaget”s [2] early work on the moral judgment of children. Like Piaget, Kohlberg [1],[3] used interview data to develop a six stage theory of moral development. Stages were believed to follow a developmental trajectory from preconventional, conventional to postconventional and individuals progressed through them in a linear fashion. The Kohlbergian moral development theory was later criticised and revised into a sociomoral stage theory by Gibbs, who removed the post-conventional stage, arguing that this level was “existential” and not consistent across cultures [4].

The sociomoral stage theory is divided into two levels: immature and mature [5]. The immature level is subdivided into Stages 1 and 2 and the mature into Stages 3 and 4. In Stage 1, moral justifications are rule-based and based upon authority or punitive consequences of rule violation. Stage 2 is the development of a superficial understanding of moral justifications arising from social interactions, for example, deciding to help others because that person may help you in the future. Stage 3 requires a prosocial understanding of emotional states (e.g. empathy), care and good conduct. At the highest level of moral reasoning, Stage 4, an understanding of the complex social structures in which we live are apparent. Justifications may be based upon constructs such as rights, values and character within society.

### 1.2. Sociomoral reasoning and antisocial behavior

Lack of progression through the immature to mature stages of moral development has been viewed as a risk factor for antisocial behaviour in adolescents and adults [6]. For example, delays in moral development significantly predict disruptive and aggressive classroom behaviour, diminished social competencies and social status [7]. In contrast, achieving a mature stage of moral reasoning (i.e. at least stage 3) by adolescence may “buffer” against delinquent behaviour [8]. However, the relationship between moral reasoning and illegal behaviour may be curvilinear rather than linear [9], as typically developing children often show fewer behavioural problems in earlier and later moral reasoning stages. In addition, internalisation of criminal sentiments (e.g. tolerance for law violation or identification with criminal others) has been found to undermine this “buffer” between mature sociomoral reasoning and delinquent behaviour [10].

### 1.3. Sociomoral reasoning and antisocial behaviour in ADHD

Children with ADHD are at increased risk for a number of negative outcomes in later adulthood with approximately one third developing significant antisocial behaviour characteristics [11]–[14]. Although antisocial behaviour is more likely for those with a diagnosis of ADHD comorbid with conduct disorder, ADHD can be a risk factor for anti-social behaviour in its own right [15]. ADHD is frequently linked to problems with peers, traffic violations and vehicle accidents [16], as well as pathological gambling, unplanned pregnancy and antisocial personality disorder [17]. The prevalence of ADHD in male prisoners is estimated to far exceed that of the general population [18] and people with ADHD frequently get fired, change jobs and have lower job performance than people without ADHD [19].

Externalising problems, such as conduct disorder, has been found to predict antisocial behaviour and adult convictions in later life [20]. Whilst Colledge and Blair [21] argued that it is the impulsivity component of ADHD rather than inattention that is associated with antisocial behaviour, it is important to consider the combined effect of all three core symptoms of ADHD (impulsivity, inattention and hyperactivity) as they combine to impair the ability of the person to cope, leading to antisocial behaviour and dysfunctional interpersonal relationships [22],[23].

Despite overall improvement of functioning as individuals with ADHD move into their third decade, antisocial behaviours remain substantially greater in those with ADHD than comparison groups [13],[14]. This may be because adults with ADHD are by definition impulsive, and therefore more likely to have difficulty anticipating the dangerous consequences of their actions [24], or alternatively they may have less mature moral reasoning. It is thought that moral reasoning relates to executive function, perhaps through internalisation of speech [25]. As such, moral reasoning is likely to be affected by the presence of a disorder such as ADHD due to the executive dysfunction common in ADHD [26]–[29]. In childhood and adolescence, moral reasoning was found to be less well developed in hyperactive-impulsive children and those with ADHD than control populations [30], although family income may moderate the effect [31].

### 1.4. Sociomoral reasoning and intelligence

In a meta-analysis by Stams et al. [8], a number of factors were identified as moderators between moral judgment and delinquency, including socioeconomic status, age, gender, intelligence, psychopathy, type of offence, institutionalisation, type of comparison group and type of measurement of moral reasoning. Furthermore, a disconnection between antisocial personality traits and sociomoral reasoning has been reported, which may be mediated by intelligence [32]. Adult offenders are generally able to reason at a mature sociomoral level, indicating that their antisocial traits may not relate to lower levels of sociomoral reasoning [33]. This incongruity between cognition and behaviour has previously been reported in offenders of average intelligence, who may show substantial differences between their reasoning and their behaviour [34]. Although greater intelligence and higher-level education reflects a greater capacity for abstract thinking, which may relate to more advanced stages of moral judgment [35], such mature moral reasoning may not be played out in behaviour.

In summary, research to date is inconclusive as to whether those with ADHD (in particular, adults with ADHD) achieve lower levels of sociomoral reasoning than those without, and also whether antisocial traits/behaviours relate directly to immature moral reasoning. There is emerging evidence that a number of factors may intervene in any such relationships, particularly that of intelligence. In this pilot study we assessed whether sociomoral reasoning in adults with ADHD is related to relevant underlying factors, including antisocial personality traits and intelligence. It was hypothesised that (H1) sociomoral scores would be negatively correlated with antisocial personality traits (i.e. those with higher sociomoral scores would exhibit fewer antisocial traits), (H2) sociomoral scores would be positively correlated with estimated full-scale IQ; and (H3) sociomoral scores would be lower in adults with ADHD than healthy controls. A further aim was to explore the relationship between antisocial personality traits, sociomoral reasoning and intellectual functioning. Two hierarchical multiple regressions were conducted to examine, first the incremental contribution of antisocial personality traits and intelligence to sociomoral reasoning, and second, the incremental contribution of sociomoral reasoning and intelligence to antisocial personality traits.

## 2. Materials and Methods

### 2.1. Participants

The sample consisted of 22 adults with ADHD, recruited from the Maudsley Adult ADHD Service, and 21 healthy controls. The ADHD Service is a UK national and specialist service, providing assessment and treatment for adults with ADHD. Inclusion criteria were aged between 18 and 65 years; IQ > 70 (i.e. no history of learning disability); no history of severe psychiatric disorder (e.g. schizophrenia, bipolar, personality disorder) or current major depressive disorder, and agreement to refrain from taking ADHD medication for 48 hours prior to the assessment. Participants were excluded if they had a primary diagnosis of substance abuse, history of autistic disorders, neurological impairment or head injury.

Healthy controls were recruited from a University volunteer database and circular email list. Controls were recruited on the basis of the inclusion and exclusion criteria described above, with the additional exclusion criteria of a diagnosis of ADHD (in childhood or adulthood).

Demographic information, together with information on ADHD subtype, was recorded from a review of clinical records. Relevant information was obtained from controls in a brief interview.

### 2.2. Measures

#### 2.2.1. Sociomoral Reflection Measure—Short Form

This is a “production measure” of moral judgment, where participants are asked to verbalise their own reasoning to questions about moral dilemmas. It includes 11 questions (e.g. “Think about when you've made a promise to a friend of yours. How important is it for people to keep promises, if they can, to friends?”). For each question the response choice is “Very important”, “Important” or “Not important” and the participant is asked to rationalise their answer. The response is scored using a set of heuristic rules detailed in the SRM manual [5]. Scoring yields a Sociomoral Reflection Maturity Score (SRMS) of between 1 and 4, pertaining to an overall global sociomoral reasoning stage. As part of the SRM-SF procedure, this is multiplied by 100, with a higher score indicating higher sociomoral reasoning.

Each SRMS can be further classified within a developmental range based on point boundaries as specified in the manual. This classification system includes the four main stages, with two transition stages between each, creating ten Global Stages, as follows: Stage 1: 100–125 (Transition stages between 1 and 2: 126–149 and 150–174); Stage 2: 175–225 (Transition Stages between 2 and 3: 226–249 and 250–274); Stage 3: 275–325 (Transition Stages between 3 and 4: 326–349 and 350–374); and Stage 4: 375–400.

The SRM-SF is reported to be a valid measure among both males and females of various age-groups, including university students, adults, and delinquent adolescents [36], and has good psychometric properties such that it is an acceptable alternative to previously developed, more time-consuming, measures [6].

For the current study, inter-rater reliability was obtained by second rater who randomly selected and blind-rated 10 questionnaires, 5 from each group respectively. Scoring agreement was within recommended parameters on all indices of inter-rater reliability in manual at *r* = 0.80 for the SRMS (achieved *r* = 0.82, *P* = 0.01). The intra-class coefficient, based on absolute agreement, was also calculated and indicated good reliability (ICC = 0.78).

#### 2.2.2. Socialisation Scale (GSS) from the California Psychological Inventory

This is a 54 item “True” or “False” scale that measures the extent to which the individual has internalised the values of society [37]. It has been shown to be a valid measure of antisocial personality traits [38]. Behaviour is ordered along a continuum from delinquent antisocial behaviours to generally accepted social behaviours and actions. Possible scores range from 0 to 54, with lower scores indicating the person is more likely to possess antisocial personality traits.

#### 2.2.3. National Adult Reading Test

The NART was administered as a measure of estimated FSIQ [39].

### 2.3. Procedure

Participants gave informed written consent to participate and were informed that results would be anonymous and confidential and would not affect their care. Data reported here was collected as part of a larger battery of measures in a study of decision-making. Participants were informed that the purpose of the study was to examine how people make decisions. Participants with ADHD were asked to refrain from taking stimulant medication for 48 hours prior to the assessment because the current assessment was administered at the same time as a study of a cognitive decision-making task that required patients to be off medication. This request has been used across various studies and there is no evidence to suggestion that discontinuation leads to withdrawal or exacerbation of symptoms [40]. Participants were texted two days before the testing session to remind them not to take their medication and all participants confirmed they had discontinued taking their ADHD medication during this period except for one, who was excluded at that stage. Participants were tested individually in a quiet room at the University with the exception of one who no longer lived locally and was tested in her home. Participants were paid £30 for participation plus travel expenses. Ethical approval was granted by the South East London Research Ethics Committee (REF: 10/H0807/34).

## 3. Results

### 3.1. Descriptive information

The ADHD group consisted of 22 participants (14 males), with a mean age of 36.9 years, and a mean of 15 years of education. Eleven had primarily inattentive subtype, eight had combined inattentive-hyperactive/impulsive subtype and three had not been classified. Twenty participants (91%) reported taking medication for ADHD (*N* = 15 Methylphenidate; *N* = 4 Dexamfetamine; and *N* = 1 Atomoxetine). The control group consisted of 21 participants, with a mean age of 35.6 years and a mean of 16 years of education. Both ADHD patients and controls were predominantly white British (16/22 and 19/21, respectively).

### 3.2. Group differences

The two groups showed no significant differences in gender (χ^2^_(1)_ = 0.30, *P* = 0.59, years of education (t_(41)_ = 1.63, *P* = 0.11), age (t_(41)_ = -0.40, *P* = 0.69), or intellectual functioning (based on estimated FSIQ from the NART; t_(41=)_ =1.02, *P* = 0.31; see Table 1). An independent t-test revealed a significant difference, and large effect size, between the groups on the GSS (t_(41)_ = 3.7, *P* < 0.001), with the ADHD group indicating significantly more antisocial personality traits (Table 1). There was no difference between the groups with regard to SRM-SF.

**Table 1.** IQ, antisocial and sociomoral reasoning scores by group.

Measure	ADHD		Controls		*t*-value	Cohen's d
	mean	SD	mean	SD		
IQ	111.55	10.13	114.57	9.30	0.31	0.31
GSS	27.32	7.19	34.33	4.96	3.7**	1.0
SRM-SF	320.32	34.74	324.44	22.21		0.14

### 3.3. Sociomoral scores

Table 2 shows the frequency with which different sociomoral (SRM-SF) level scores were obtained for the ADHD and control groups. The pattern of scores is similar for the two groups with most scores falling at Stage 3 and the transition stage from 3 to 4. Four participants (1 control and 3 ADHD) were excluded from the analysis as fewer than seven of the eleven questions were scorable (e.g. they provided limited responses such as “because it is important” with no elaboration).

**Table 2.** Frequency of SRM-SF global stages for the ADHD and control groups.

SRM-SF global stage	ADHD (*N* = 22)	Controls (*N* = 21)	Both (*N* = 43)
Unscorable	3 (13.6)	1 (4.8)	4 (9.3)
Stage 1	-	-	-
Transition 1-2 (1)	-	-	-
Transition 1-2 (2)	-	-	-
Stage 2	-	-	-
Transition 2-3 (1)	-	-	-
Transition 2-3 (2)	3 (13.6)	1(4.8)	4 (9.3)
Stage 3	6 (27.2)	9 (42.8)	15 (34.9)
Transition 3-4 (1)	7 (32.0)	8 (38.1)	15 (34.9)
Transition 3-4 (2)	3 (13.6)	2 (9.5)	5 (11.6)
Stage 4	-	-	-

n(%).

SRM-SF: Sociomoral Reflection Measure–Short Form.

### 3.4. Correlations

Table 3 lists the results from binary logistic regression on assessing predictors of confidence to know when to get medical care and when I can handle myself. Married men are 50% more likely than unmarried men to agree they are confident in knowing when to get medical care (*OR*: 1.47, *CI:* 1.04−2.08). Uninsured men are 56% less likely than men with public insurance to agree they are confident in knowing when to get medical care (*OR:* 0.44, *CI:* 0.25−-0.77). Men in good and very good/excellent health were 1.57 and 2.11 times more likely to agree they are confident in knowing when to get medical care than those in poor health (*OR:* 1.57, *CI:* 1.07−2.29 and *OR:* 2.11, *CI:* 1.39−3.18).

#### 3.4.1. Sociomoral reasoning and antisocial behavior

For the whole group (*N* = 43), a significant positive correlation was found between the SRM-SF and the GSS (*r* = 0.35, *P* < 0.05). When the groups were analysed separately, SRM-SF and GSS was significantly correlated for the ADHD group only (*r* = 0.44, *P* < 0.05). See Table 3 for results of correlations by group.

**Table 3.** Correlations between measures for the two groups (ADHD above the diagonal, and controls below).

	IQ	GSS	SRM-SF
IQ	-	-0.07	0.59**^a^
GSS	-0.08	-	0.44^*a^
SRM-SF	0.41* ^a^	0.21^a^	-

GSS: Gough Socialisation Scale total; SRM-SF: Sociomoral Reflection Measure total; IQ: NART estimated FSIQ; ^a^ One-tailed in relation to specific hypotheses. *P < 0.05, **P < 0.01.

#### 3.4.2. Sociomoral reasoning and intelligence

For the whole group (*N* = 43), a significant positive correlation was found between the SRM-SF and predicted FSIQ (*r* = 0.52, *P* < 0.001). When the groups were analysed separately, SRM-SF and predicted FSIQ was significantly correlated for both the ADHD group (*r* = 0.59, *P* < 0.01) and control group (*r* = 0.41, *P* < 0.05). See Table 3 for results of correlations by group

### 3.5. Hierarchical multiple regressions

In order to investigate the incremental contribution of antisocial personality traits and intelligence to sociomoral reasoning, a multiple regression using a hierarchical (blockwise) entry method was conducted on the data (*N* = 39) (Table 4). Age, gender and diagnostic group (i.e. ADHD/non-ADHD classification) were entered in the first block to account for their possible effects; the GSS score for antisocial traits was added in the second block, followed by the NART estimated FSIQ in the third block.

**Table 4.** Summary of hierarchical regression predicting sociomoral reasoning for the combined ADHD and control groups (*N* = 39).

Model		*B*	Std. Error	*β*	*t*-value	Adjusted R squared
1	(Constant)	310.42	18.05		17.20***	-0.06
	Age	0.39	0.46	0.14	0.83	
	Gender	0.30	9.95	0.01	0.03	
	Diagnostic	-4.23	9.53	-0.08	-0.44
	Group					
2	(Constant)	230.41	34.37		6.71***	0.10
	Age	0.70	0.44	.25	1.57	
	Gender	-2.39	9.24	-0.04	-0.26	
	Diagnostic	8.46	10.00	0.15	0.85	
	Group					
	GSS	2.04	0.77	0.48	2.66*
3	(Constant)	74.25	51.28		1.45	0.34
	Gender	2.99	8.01	0.05	0.37	
	Age	-0.01	.42	-0.00	-0.02	
	Diagnostic	12.84	8.61	0.23	1.49	
	Group					
	GSS	1.86	0.65	0.44	2.84**	
	FSIQ	1.61	0.44	0.56	3.71***

*P < 0.05; **P < 0.01; ***P < 0.001.

Models 1 and 2 were not significant (*P* = 0.83 and *P* = 0.11, respectively) but Model 3 was significant (*P* < 0.01). In Model 1, none of the predictors were significant and in Model 2, only the GSS score for antisocial traits was significant (*P* < 0.05), which explained 10% of the variance in sociomoral reasoning. In Model 3, both the GSS score and IQ were significant predictors (*P* < 0.01, *P* < 0.001, respectively) of sociomoral reasoning; the model as a whole accounting for 34% of the variance in moral reasoning.

We repeated the multiple regression with the GSS as the dependent variable in order to investigate the incremental contribution of sociomoral reasoning and intelligence to antisocial personality traits. Age, gender and diagnostic group (i.e. ADHD/non-ADHD classification) were entered in the first block to account for their possible effects; the SRM-SF total was added in the second block, followed by the NART estimated FSIQ in the third block (see Table 5).

**Table 5.** Summary of hierarchical regression predicting antisocial behaviour for the combined ADHD and control groups (*N* = 39).

Model		*B*	Std. Error	*β*	*t*-value	Adjusted *R* squared
1	(Constant)	39.291	3.682		10.67***	0.21
	Age	-0.152	0.094	-0.233	-1.61	
	Gender	1.322	2.029	0.095	0.65	
	Diagnostic Group	-6.230	1.944	-0.467	-3.21**
2	(Constant)	13.000	10.445		1.25	0.32
	Age	-0.185	0.088	-0.283	-2.10*	
	Gender	1.297	1.873	0.093	.69	
	Diagnostic Group	-5.872	1.799	-0.440	-3.26**	
	SRM-SF	0.085	0.032	0.360	2.66*
3	(Constant)	19.742	12.140		1.63	0.33
	Age	-0.138	0.098	-0.211	-1.41	
	Gender	0.871	1.909	0.063	.46	
	Diagnostic Group	-6.230	1.825	-.467	-3.41**	
	SRM-SF	0.106	0.037	0.449	2.84**	
	FSIQ	-0.131	0.121	-0.194	-1.08

*P < 0.05; **P < 0.01; ***P < 0.001.

Models 1, 2 and 3 were all significant (*P* < 0.05, *P* < 0.001 and *P* < 0.01 respectively). In Model 1, only group was significant (*P* < 0.01), accounting for 21% of the variance in antisocial behaviour. In the Model 2, age, group and sociomoral reasoning were significant (*P* < 0.05, *P* < 0.01, *P* < 0.05 respectively), explaining 32% of the variance in antisocial behaviour. Adding NART estimated FSIQ in Model 3 did not improve the amount of predictive variance in antisocial behaviour already explained by age, group and sociomoral reasoning.

## 4. Discussion

This pilot study investigated the relationship between antisocial personality traits, intelligence and sociomoral reasoning in adults with ADHD. A significant negative correlation between sociomoral reasoning and antisocial personality traits was found for the group as a whole and the ADHD group, supporting (H1). The hypothesis of a significant positive correlation between sociomoral reasoning and intelligence (H2) was supported for both groups. The hypothesis that adults with ADHD would obtain lower sociomoral scores than healthy controls (H3) was not supported. The two groups were matched for gender, age and estimated IQ. Results from the first hierarchical multiple regression indicated that when controlling for age, gender and diagnostic classification, both antisocial traits and IQ were significant predictors of sociomoral reasoning, with IQ proving the most powerful predictor. Indeed, IQ added 24% to the variance of sociomoral reasoning above and beyond that of antisocial personality traits. This suggests that IQ has a unique variance in relation to sociomoral reasoning, which should be addressed in future research. Results from the second hierarchical multiple regression indicated that both diagnostic group and sociomoral reasoning were significant predictors of antisocial traits, explaining a similar amount of variance in the final model. Therefore, both appear to be important in explaining the amount of antisocial personality traits among the participants.

As hypothesised, intelligence was strongly associated with sociomoral reasoning perhaps reflecting that intellectual function plays a protective role in the development of mature moral reasoning. The finding supports previous research which reported that intellectual functioning was an important mediator between sociomoral reasoning and illegal behaviour [32]. Contrary to prediction, sociomoral reasoning did not significantly differentiate between the two groups. Whilst most participants (in both groups) achieved at least Stage 3 of moral development (hence their sociomoral reasoning would be considered mature), the ADHD group obtained lower sociomoral reasoning scores compared with controls but higher than those reported previously in mentally disordered offenders [32]. In a broader context, these findings suggest that sociomoral development may relate to antisocial traits/behaviour, although causality has not been established. Hence the relationship between sociomoral reasoning and prosocial development is complex and requires further research. Disruption to social relationships in young people with ADHD has been well documented and perhaps the difficulty lies less in the capacity for sociomoral reasoning and its development, and more in its pragmatic translation into social skills and activities in daily life [41].

Some confounding variables must be acknowledged which were not controlled for in the current study, such as ADHD subtype. As this was a pilot study, the sample size was small. A larger sample size would have allowed the division of subgroups (predominantly inattentive, hyperactive/impulsive or combined) to assess the relative impact of each on sociomoral reasoning and antisocial personality traits. Whilst participants refrained from taking their medication for 48 hours prior to the assessment, this is unlikely to have unduly influenced their responses to the sociomoral assessment which draws on an individual”s experiential understanding of the world and social values. However the sample were recruited from clinic referrals and most had a history of treatment with ADHD medication; their experiences (and hence their sociomoral reasoning) may differ from epidemiological samples and those who have not had access to treatment. The assessment relied on self-report methods; hence it is possible that antisocial traits and/or sociomoral responses were not reliably reported. However the “production measure” presentation style of the SRM-SF reduces the risk of socially desirable responding as the individual is asked to expand and rationalise their answers.

These findings may have important implications for treatment. In the current pilot study, those attending an ADHD outpatient clinic reported significantly greater antisocial traits compared with the normal population. It is important that psychological treatments are provided to address both ADHD and comorbid problems [42]. A strong treatment effect has been reported in a community-run randomised controlled trial evaluating a cognitive skills programme that targets antisocial attitudes and behaviour, sociomoral reasoning and social problem-solving skills in youths and adults with ADHD [43]. Treatment should particularly address the needs of individuals with low IQ scores (e.g. those with borderline and mild learning disability) who may be more at risk of developing a less sophisticated and mature understanding of sociomoral values [44].
